# Differentiation of Fat-poor and Atypical Adrenal Adenomas from Metastases: MRI-based Radiomic, Radiologic, and Radiomic-radiologic Machine Learning Models

**DOI:** 10.2174/0115734056399745260330003149

**Published:** 2026-04-08

**Authors:** Ceyda Turan Bektaş, Hasan Bulut, Ece Ateş Kuş, Melis Baykara Ulusan, Abdullah Soydan Mahmutoğlu

**Affiliations:** 1 Department of Radiology, Istanbul Training and Research Hospital, Istanbul, Türkiye; 2 Department of Radiology, Ankara Etlik City Hospital, Ankara, Türkiye; 3 Department of Diagnostic and Interventional Radiology, Medical Faculty OWL, University of Bielefeld, Klinikum Lippe, Bielefeld, Germany; 4 Nottingham Breast Institute, Nottingham University Hospitals, NHS Trust, Nottingham, UK

**Keywords:** Adenoma, Metastasis, Artificial intelligence, Machine learning, Texture analysis, Radiomics

## Abstract

**Introduction:**

The accurate differentiation of fat-poor and atypical adrenal adenomas from metastases remains a diagnostic challenge. This study aimed to evaluate the predictive value of MRI-based radiomic, radiologic, and combined radiomic-radiologic machine learning (ML) models.

**Methods:**

This single-center retrospective study included 37 patients with 44 adrenal masses (19 adenomas; 25 metastases). Data were split into training and testing sets (2:1). To expand the training set, data augmentation was performed by multiple sampling (56 labeled slices from 30 masses). Radiomic features were extracted from T2-weighted (T2W), in-phase, out-of-phase, and apparent diffusion coefficient (ADC) sequences, while mass size, T2W signal intensity, heterogeneity, and signal drop were assessed as radiologic features. Dimension reduction was performed by reliability analysis and wrapper-based feature selection with five algorithms. A support vector machine (SVM) was used for classification, and performance was assessed using 10-fold cross-validation and unseen testing. Friedman test and post-hoc analyses compared bootstrapped unseen test area under the curve (AUCs).

**Results:**

Only 12% of radiomic features demonstrated excellent reproducibility. A significant difference was observed among the three models, χ^2^(2)=779.5, *p*<0.001. The combined radiomic-radiologic model achieved the best performance (AUC 0.939; accuracy 85.7%), outperforming radiomic-only (AUC 0.898; accuracy 85.7%) and radiologic-only (AUC 0.857; accuracy 78.5%) models (adjusted *p*<0.001).

**Discussion:**

Integrating radiomic and radiologic features improved classification performance compared to using either feature set alone. Although the reproducibility of radiomic features was limited, their complementary value enhanced model robustness.

**Conclusion:**

A combined radiomic-radiologic ML model based on multi-sequence MRI may serve as a promising non-invasive tool for differentiating atypical adrenal adenomas from metastases.

## 1 INTRODUCTION

The increasing use of cross-sectional imaging has led to the incidental detection of adrenal lesions in up to 5% of patients [1, 2]. While most of these are benign adenomas, metastases remain the most common malignant lesions.

Lipid-rich adenomas are easily diagnosed using CT or MRI. However, lipid-poor or atypical adenomas often appear indeterminate on unenhanced CT or chemical shift imaging (CSI) [3–5], necessitating adrenal washout CT or even biopsy [6]. Some metastases mimic adenomas in imaging due to overlapping enhancement or heterogeneity [7–10]. Accurate differentiation is vital for oncologic management, where imaging plays a key role [11].

Radiomics enables the extraction of quantitative imaging features, providing insights beyond visual interpretation [12, 13]. Recent studies have applied radiomics in adrenal lesion classification, but often with limited modalities or without radiologic context [14-16].

Unlike prior studies using CT or single-sequence MRI, we developed a multimodal ML model integrating radiomic and radiologic features extracted from T2W, in-phase, out-of-phase, and ADC sequences.

Romeo *et al.* achieved 80% accuracy using texture features from limited MRI sequences without radiologic inputs or external testing [4]. Tu *et al.* analyzed only T2W first-order features. Our hybrid model outperformed both with 85.7% accuracy and an AUC of 0.939, using bootstrapped testing and direct model comparisons [5].

CT-based studies like Shi *et al.* reported moderate performance (AUC ~0.77) with limited reproducibility due to low soft tissue contrast [17]. Our MRI-based approach, especially with functional ADC input, offers superior lesion characterization.

To distinguish fat-poor or atypical adenomas from metastases, alternative imaging methods, long-term follow-up, biopsy, or surgical resection are often required [4].

We propose this MRI-based ML model as a non-invasive decision support tool to improve diagnostic confidence and workflow efficiency.

## 2 MATERIALS AND METHODS

### 2.1 Ethics

The study was approved by Clinical Research Ethics Committee of University of Health Sciences Istanbul Training and Research Hospital, Türkiye. The review board of our institution approved this single-center retrospective study and waived the requirement for obtaining informed consent.

### 2.2 Study Design

Patients were selected from our picture archiving and communication system (PACS) covering the period from June 2015 to December 2019. Upper abdominal MR reports in this time span that include “adenoma” or “metastasis” words were scanned retrospectively, and 339 adrenal masses were detected.

Inclusion criteria were as follows: (i), masses with histopathological diagnosis, (ii), patients with negative clinical situation and negative hormone profile for pheochromocytoma, (iii), stable masses without histopathologic diagnosis at least 1 year CT or MRI follow-up (were accepted as adenomas), (iv), in oncologic patients; newly developing adrenal masses or masses with an increase in size over 20% within 6 months or masses with PET-CT findings compatible with metastasis (were accepted as metastases), (v), atypical adenomas that have radiologic features such as heterogeneous appearance or heterogeneous signal-intensity drop on opposed-phase CSI, large size, the presence of internal cystic changes or necrosis (10).

The exclusion criteria were as follows: (i), adrenal lesions smaller than 1 cm in long axis, (ii), prominently hemorrhagic masses, (iii), masses with marked or purely cystic components, (iv), typical fat-rich adenomas which are smaller than 4 cm, homogeneous, have fine margins, lipid content and low-intermediate signal in T2W images (10), (v), suboptimal images with respiratory or movement artifacts, (vi), histopathologically diagnosed nonadenoma and nonmetastatic malign adrenal tumors.

Finally, according to these inclusion and exclusion criteria, the final study group comprised 37 patients with 44 adrenal masses.

Masses were semi-randomly assigned to the training and testing datasets with a ratio of 2:1. To prevent potential information leakage, patients with bilateral masses were included only in the training set before the random split. The other patients with single masses were randomly assigned to the training and testing sets. To increase training data volume, the training data was augmented with multiple samplings (please see the “segmentation” section of “methods” for details). The test data was processed separately and remained completely unseen by the algorithms until the final performance evaluation.

Given the relatively small sample size in this study (n = 37 patients, 44 adrenal masses), which may limit the learning capacity and generalizability of machine learning (ML) algorithms, we employed a data augmentation strategy to enhance the training dataset.

Specifically, a multi-slice augmentation approach was implemented, in which all artifact-free axial slices containing visible tumor tissue were manually segmented and included in the training phase. This strategy resulted in 56 labeled slices from 30 masses, thereby increasing both the diversity and volume of training data while maintaining clinical and radiological plausibility.

To minimize potential bias from within-subject redundancy, patients with bilateral adrenal lesions were included only in the training set, and no patient contributed data to both the training and test sets. The test set was kept entirely independent and remained unseen during training and feature selection.

Although slice-based augmentation enhances the number of training instances, it may introduce bias if not properly controlled. Therefore, strict patient-level separation and bootstrapped evaluation on independent test cases were employed to ensure model robustness and reduce the risk of overfitting.

To give a simplified overview to the readers, the technical study pipeline is given in Fig. (**1**).

### 2.3 MRI Technique

All MRI examinations were performed on a 1.5T MRI scanner (SignaHDxt, GE Medical Systems, Milwaukee, Wisconsin, USA) with routine upper abdomen MRI sequences [18]. Patients underwent imaging in a supine position, and all MR images were performed with the dedicated 8-channel body array coil in the transverse section. Single breath-hold axial 2D gradient-echo T1-weighted sequence (CSI), T2W fast spin echo imaging, and echo planar imaging (EPI) diffusion weighted imaging (DWI) sequences (b=0,800 s/mm2) were used. ADC maps for each lesion were generated from DWI obtained using b0 and b800 values, using software (Functool) on a separate workstation (Advantage Workstation 4.4-GE Medical Systems). The settings were as follows; for *out-of-phase sequence* repetition time/echo time (TR/TE); 115-255 /2.01-2.25 ms, field of view (FOV); 780-900 X 380-440 mm, slice thickness;5.5-7 mm, number of excitations (NEX): 1; for *in-phase sequence* TR/TE; 130-255/: 4.2-4.81 ms, FOV; 780-900 x 380-440 mm, slice thickness; 5.5-7 mm, NEX: 1; for *T2W sequence* TR/TE:613-905/87-91 ms, FOV; 780-900 x 380-440 mm, slice thickness; 5.5-7 mm, NEX: 0.55; for *DWI sequence* TR/TE; 5425-6025 / 64-66 ms, FOV; 820-900 X 380-440 mm, slice thickness;5.5-7 mm, NEX: 1-2.

### 2.4 Image Preprocessing

All MRI sequences underwent N4 bias field correction to remove non-uniform low-frequency intensity values [19].To minimise differences in intensity range, all images were normalized by centering the voxel image intensity values at the mean with the standard deviation [20]. The intensity normalization scale was set to 1. Pixel spaces in all image slices were rescaled to an in-plane resolution of 1x1 mm^2^ [21]. As recommended by developers of PyRadiomics software, grey-level discretisation was done based on a fixed bin-width method with values of 0.02 for in-phase, out-of-phase, and T2W images and 0.03 for ADC [22, 23].

### 2.5 Segmentation

The masses were manually segmented from all four MRI sequences using 3D-Slicer software (version 4.8). Segmentations were independently performed by two experienced abdominal radiologists to assess the reliability of radiomic features. In training data, all available slices of the masses were segmented to augment the data set in the training phase, as long as the slices have no quality issues, such as artefacts. In the test data set, the largest cross-sectional area of the tumours was selected for the segmentation. Segmentation style is presented in Fig. (**2A**-**H**).

### 2.6 Radiomic Feature Extraction

Radiomic features were extracted using PyRadiomics software (Python 2; PyRadiomics 1.3; Numpy 1.13; SimpleITK 1.1; PyWavelet 0.5) [24]. The features were extracted from one original, three filtered, and five wavelet-transformed images. The Laplacian of Gaussian (LoG) filter was used for image filtration with values of 2 mm for fine patterns, 4 mm for medium patterns, and 6 mm for coarse patterns. The extracted features were as follows: *(i)*, 18 first-order features; *(ii)*, 14 grey-level dependence matrix; *(iii)*, 23 grey-level co-occurrence matrix; *(iv)*, 16 grey-level run-length matrix; *(v)*, 16 grey-level size zone matrix; and *(vi)*, five neighboring grey-tone difference matrix features. Ninety-two unique features were extracted from nine different image types, adding up to 828 features per MRI sequence and 3312 features from four MRI sequences per patient. Detailed descriptions and mathematical formulas for these features have been described in website of the software (https://pyradiomics.readthedocs.io).

### 2.7 Radiologic Feature Extraction

Radiological features were extracted from conventional sequences (T2W, in-phase, and out-of-phase sequences) by an experienced abdominal radiologist. Based on the literature, T2W signal intensity, presence of signal drop in out-of-phase sequence in reference to in-phase sequence, and heterogeneity of the masses in any sequence were evaluated. Signal intensity index (SII) values were measured from an ellipsoid ROI, which included 2/3 of each mass, and the CSI dataset was created using the following formula (1) [25, 26]:

SII= (signal on in-phase MRI - signal on out-of-phase MRI) /(signal on in-phase MRIX100)…. (1)

T2 signal evaluation was done according to the muscle signal as hyperintense, hypointense, or isointense. In addition to the imaging appearances, mass size was also included in radiological features. The longest diameter was measured for each lesion in axial T2 images.

### 2.8 Reliability Analysis

Using the segmentation data by two experienced abdominal radiologists, a reliability analysis was conducted to select radiomic features with excellent reproducibility. Intra-class correlation coefficients (ICC) were calculated for each radiomic feature. The features with an ICC of ≥0.9, which indicates “excellent” reproducibility, were included in the further dimension reduction steps [27].

Of the 3,312 radiomic features extracted from four MRI sequences, 402 (12.1%) demonstrated excellent interobserver reproducibility, with intraclass correlation coefficients (ICC) ≥ 0.9. A considerable proportion of the remaining features showed moderate to good reproducibility, with ICC values ranging between 0.5 and 0.9.

The detailed distribution of ICC values across all extracted features is provided in Table **1**. To ensure robustness against inter-reader variability, only features with ICC ≥ 0.9 were retained for subsequent feature selection and model development.

All segmentations were independently performed by two experienced abdominal radiologists, with consensus achieved in cases of disagreement.

### 2.9 Feature Selection Experiments

Feature selection was performed using multiple methods as detailed in the supplementary material (**Supplementary Material, Part A**).

The wrapper-based classifier-specific feature selection and model optimisation were performed using WEKA toolkit version 3.8 (University of Waikato) [28].

In the training phase, a 10-fold cross-validation approach was adopted. Features with at least 2-fold cross-validation were included in the outer loop for final model development. For radiomic and radiologic features, 5 different feature ranking methods were used within a wrapper evaluator [29]. The search technique was incremental wrapper-based subset selection *via* replacement and early stopping [30]. The feature ranking methods were as follows: correlation attribute evaluator, information gain attribute evaluator, ReliefF, significance attribute evaluator, and symmetric uncertainty attribute evaluator. The correlation attribute evaluator assesses the worth of a feature by quantifying the Pearson correlation between the feature and the respective classes. Information gain attribute evaluator assesses the worth of a feature by quantifying the information gain with respect to the classes, without considering feature interactions. ReliefF ranks the features according to k-nearest neighbors. The significance attribute evaluator assesses the features by calculating the probabilistic significance as a two-way function. The symmetric uncertainty evaluator assesses the feature importance by calculating the symmetrical uncertainty related to another set of features.

### 2.10 Machine Learning-based Classifications

Machine learning-based classifications were performed using the WEKA toolkit. Firstly, radiomic and radiologic only models were created with features selected by different algorithms. Then, the best feature subsets were also combined to form a radiomic-radiologic model. A support vector machine (SVM) was used in classifications. Parameters of the support vector machine classifier were as follows: c, 1; calibrator, logistic; kernel, polykernel; and tolerance, 0.001. Further parameter tuning (C, kernel type, tolerance parameters) was also performed following wrapper-based feature selection for the best model. Considering the potential inherent bias introduced by the internal validation techniques, the performance evaluation was done both in the training data as validation and in the unseen data as testing. The performance of the algorithms was mainly evaluated with the area under the receiver operating characteristic curve (AUC). In addition, the accuracy, sensitivity, and specificity were also calculated for further evaluation. Using predicted probabilities, receiver operating characteristic (ROC) curves were drawn, and bootstrapped AUCs were calculated with 1000 iterations. Considering the distribution of the bootstrapped AUCs, comparison of the model performance was done using the Friedman test, and if this test was significant, further pairwise post-hoc analysis was performed. Bonferroni correction was applied in the pairwise analyses. A two-tailed adjusted *p*-value less than 0.05 indicated statistical significance.

## 3 RESULTS

### 3.1 Baseline Characteristics

A total of 37 patients with 44 adrenal masses met the eligibility criteria. Thirty lesions (56 labeled slices) were assigned to the training set, and 14 lesions (14 labeled slices) to the test set. Detailed patient, lesion characteristics, and conventional radiological features are presented in Table **2**.

### 3.2 Reliability of Radiomic Features

Among 3312 radiomic features extracted from four MRI sequences, 402 (12%) showed excellent interobserver reproducibility. These included 242 from ADC, 98 from out-of-phase, 36 from T2W, and 26 from in-phase images. Only these reproducible features were used in subsequent feature selection analyses.

### 3.3 Feature Selection Experiments

The feature selection process is summarized in Fig. (**3**). For radiomic feature selection, the best performance on unseen test data was achieved using a subset of imaging features statistically associated with the final diagnosis, derived from all four MRI sequences (T2W, in-phase, out-of-phase, and ADC). In contrast, feature subsets derived from single MRI sequences or those excluding ADC generally resulted in inferior test performance.

In the case of radiologic features, three different selection approaches consistently identified the same subset as optimal. The final radiomic and radiologic features are listed in detail in Table **3**, with their distribution shown in Fig. (**4**) and their relative importance illustrated in Fig. (**5**).

Feature selection was conducted using a machine learning–based approach designed to identify the most informative features for the classification task. Several ranking techniques were applied, including methods that evaluate each feature’s statistical relevance to the diagnosis, its ability to separate similar *versus* dissimilar cases, and its overall contribution to predictive accuracy.

The selection process followed an incremental, stepwise strategy that stopped automatically when further additions no longer improved model performance. This helped optimize diagnostic performance while reducing the risk of overfitting.

To enhance the clinical interpretability of our model, the most relevant radiomic features were reviewed in detail. Notably, Run-Length Non-Uniformity (RLNU) emerged as a top-ranking feature, likely capturing the internal heterogeneity of metastatic lesions-such as necrosis or hemorrhage-compared to the more uniform structure of adrenal adenomas. Similarly, gray-level co-occurrence matrix (GLCM), Entropy and gray-level size-zone matrix (GLSZM), which measure texture complexity and heterogeneity, were consistently elevated in metastatic lesions. Additional informative features included first-order Kurtosis, indicating the peakedness of intensity distribution, and neighbouring gray tone difference matrix (NGTDM), Coarseness, which reflects the smoothness of signal transitions. These biologically and radiologically meaningful features support the model’s ability to detect clinically relevant imaging patterns, moving beyond traditional “black-box” machine learning outputs.

### 3.4 Classifications

To ensure robust model development and reliable performance assessment, we carefully addressed class balance between adenomas and metastases during data partitioning. Specifically, the training dataset included 30 adrenal masses (13 adenomas and 17 metastases), while the testing dataset comprised 14 adrenal masses (6 adenomas and 8 metastases).

This near-balanced distribution in both sets helped minimize the risk of class imbalance bias and supported a more reliable evaluation of model generalizability. Importantly, the test dataset was kept completely independent and remained unseen throughout all stages of training and feature selection.

The best radiomic model, using seven multi-sequence features, achieved an AUC of 0.898 (bootstrapped median 0.907; 95% CI, 0.902–0.913) and an accuracy of 85.7%. The best radiologic model, based on two conventional features, yielded an AUC of 0.857 (median 0.867; 95% CI, 0.860–0.875) and an accuracy of 78.5%.

Combining radiomic and radiologic features improved overall performance, with an AUC of 0.939 (median 0.948; 95% CI, 0.944–0.955) and an accuracy of 85.7%. Further parameter tuning did not yield additional improvement (Table **4**).

According to the Friedman test, differences in AUCs among the three models were statistically significant (χ^2^(2) = 779.5, *p* < 0.001). Pairwise post hoc comparisons confirmed significant differences between all model pairs (*p* < 0.001), with the combined model (mean rank: 2.59) outperforming both the radiomic-only (1.96) and radiologic-only (1.45) models. ROC curves and AUC distributions are shown in Fig. (**6A**-**D**).

## 4 DISCUSSION

### 4.1 Overview

In this work, we investigated the predictive value of machine learning in differentiating fat-poor and atypical adrenal adenomas from metastases. Following many feature selection experiments we developed, validated, and tested various MRI-based radiomic, radiologic, and radiomic-radiologic ML models. The combined radiomic-radiologic model achieved better predictive performance than radiomic-only and radiologic-only models did. This finding is consistent with prior multimodal modeling studies in other domains, where data fusion approaches, such as variational dynamic fusion using Gaussian mixtures, have shown superior performance over unimodal models [31].

More than four-fifths of the masses can be correctly and non-invasively diagnosed with this method. This evaluation on previously unseen test cases reflects principles of causal meta-generalization, which aim to ensure model robustness and transferability across varying clinical data domains [32].

Radiomic feature subsets extracted from all available MRI sequences (T2W, in-phase, out-of-phase, and ADC) resulted in a better performance than the subsets from individual (T2W only, in-phase only, out-of-phase only, or ADC only) or other combinations of sequences (all conventional sequences without ADC) did. MRI was performed using available sequences with parameters detailed in the supplementary material (**Supplementary Material, Part B**). Only less than one-fifth of the radiomic features showed excellent reproducibility.

Given the high dimensionality of radiomic features and the limited sample size, the risk of overfitting is a valid concern. To address this, we implemented several mitigation strategies, including strict feature reproducibility filtering (ICC ≥ 0.9), wrapper-based feature selection, and cross-validation during model training. Furthermore, an independent test set-kept entirely separate from the training and feature selection phases-was used for final model evaluation. Despite these precautions, the possibility of residual overfitting cannot be completely ruled out. Future studies with larger, multi-center datasets and the use of advanced regularization techniques are warranted to further reduce this risk and enhance model generalizability.

### 4.2 Previous Works

There are a few studies evaluating the role of radiomics in the characterization of adrenal masses in MR images. To date, as far as we know, there has been no study that evaluates T2, CSI, and ADC maps together in discriminating between adrenal adenomas and metastasis.

Ho *et al.* evaluated the differentiation of 23 adrenal nodules by texture analysis of enhanced and unenhanced CT and SII of CSI MRI. They found that texture analysis of unenhanced CT and CSI showed a lower diagnostic performance compared with unenhanced CT attenuation and the SII. Texture analysis of CSI did not appear to provide any further advantage over SII for distinguishing lipid-poor adenomas from malignant adrenal lesions. However, CSI alone showed higher statistical significance than the texture analysis of CSI [3]. In our study, more important radiologic - radiomic features than CSI alone were found in adenoma-metastasis differentiation.

Tu *et al.* studied first-order texture analysis (histogram) features and evaluated the combination of T2W SII and T2W heterogeneity subjectively and quantitatively (through T2W entropy). Logistic regression models can differentiate adrenal metastases from benign lipid-poor adrenal adenomas [5].

Romeo *et al.* found that the machine-learning algorithm using texture-derived features extracted from unenhanced MR images was useful in characterizing adrenal lesions as compared with a senior radiologist [4]. However, their non-adenoma study group was heterogeneous, unlike ours, and consisted of metastasis, adrenocortical carcinoma, and pheochromocytoma. They used only dual-echo and T2W images. Also, unlike in our study, radiologic-radiomic ML methods were not evaluated statistically. They considered that lesion size is a reliable parameter to classify non-adenoma lesions. In our study, although the size of the lesion is one of the most diagnostic features, there were more effective radiomic features.

Umanodan *et al.*, performed histogram analysis on ADC maps only and found that ADC histograms were useful in distinguishing adenoma and pheochromocytoma [33]. In our study, we used dual echo, T2, and ADC maps to distinguish fat-poor and atypical adenoma from metastasis, and we obtained features with high diagnostic performance from all 3 kinds of image series. This result supports the importance of the radiomic properties of ADC maps in adrenal tumor classification and revealed that studies involving larger case series and sequences are required. A recent study investigating breast cancer demonstrated that ADC-based radiomic features, combined with generalized linear modeling, could successfully predict Ki-67 expression levels, highlighting the potential of ADC maps in radiomics-based diagnostic modeling [34].

Shi *et al.* performed CT texture analysis (CTTA) on both unenhanced and contrast-enhanced images for differentiating metastasis from adenomas and found that CTTA on contrast-enhanced images provides much more useful information. As for diagnostic performance, specificity and sensitivity were 77.2% and 77.4%, respectively [17].

Zhang *et al.*, using CTTA, showed that lipid-poor adenomas are typically homogeneous, while pheochromocytomas are more heterogeneous [35]. They performed first-order texture parameters on both unenhanced and contrast-enhanced images and found that CTTA on contrast-enhanced images did not seem to provide as much useful information as it did on unenhanced images.

Yu *et al.*, using portal venous phase CTTA, found that entropy and standard deviation have high sensitivity and specificity in benign-malignant differentiation of adrenal masses in a heterogeneous group of benign and malignant adrenal masses [36]. So, the contrast effect on CT texture analysis is unclear, and future studies are required to confirm that.

### 4.3 Practical Implications and Future Directions

The most common adrenal masses in the general population are benign adenomas [37]. However, radiologists need to rule out other possibilities such as pheochromocytoma, adrenocortical carcinoma, and metastasis. Especially in the oncological patient group, differentiation of adrenal lipid-poor adenoma, atypical adenoma, and metastasis plays a critical role in determining the prognosis and treatment approach. In most cases, additional procedures such as CT-wash out, PET-CT, biopsy, or surgery are required, and the risk of morbidity due to radiation exposure, contrast agent use, and/or interventional procedures such as biopsy increases.

The diagnostic efficiency of MRI sequences other than CSI in radiological evaluation is controversial. In T2W images, adenomas are generally homogeneous and have iso-hypointense signal characteristics compared to muscle and liver, but large and atypical adenomas may appear heterogeneous [38-40]. Also, heterogeneous microscopic fat in adrenal nodules is generally benign; however, in patients with an oncologic history, rarely, microscopic fat containing a collision tumor or a microscopic fat-containing metastasis must be kept in mind [41, 42]. Quantitative ADC values of adenomas and non-adenomas show overlap, so differentiation cannot be made by DWI [43]. MR sensitivity for adenomas measuring 10–20 HU by unenhanced CT is nearly 100%, while that for lipid-poor adenomas measuring greater than 30 HU is significantly lower (13–75%) [38, 44].

It can be assumed that studies with only fat-poor adenomas and metastases will play a more effective role in clinical practice. Prospective multi-center studies with VOI, which includes the whole tumor, and an increased number of patients, will increase ML performance.

In clinical workflow, the proposed machine learning model could function as a decision support tool, particularly in cases where adrenal lesions show indeterminate or atypical imaging characteristics. Rather than replacing radiologist interpretation, the model is intended to assist in risk stratification by providing a non-invasive, quantitative second opinion-especially for oncologic patients where distinguishing benign adenomas from metastases significantly affects management decisions. Its retrospective application could also facilitate multidisciplinary discussions or help prioritize lesions for follow-up, biopsy, or further imaging. Therefore, integration of such a model into the radiology workflow holds the potential to optimize diagnostic confidence, reduce unnecessary interventions, and improve patient outcomes.

Recent studies have highlighted the feasibility of integrating radiomics models into PACS and radiologist-in-the-loop (RIL) workflows. Hawkins *et al.* [45] presented a modular system enabling real-time ML deployment within PACS for radiologist feedback. Similarly, Purkayastha *et al.* [46] integrated AI tools into open-source RIS platforms to support interactive use. Our model could be similarly adapted as a decision-support tool within clinical systems, enabling radiologist oversight and fostering practical applicability.

### 4.4 Limitations

Our study has a number of limitations that need to be highlighted. First, the number of patients was small for ML-based analysis. For this reason, we tried to augment the training data set with a multiple sampling strategy to increase the training data volume. It is also important to emphasize that test data were not over-sampled and included different patient data sets, providing completely unseen data for testing. Another limitation of our study is the use of 2D segmentation rather than 3D volumetric segmentation. While three-dimensional convolutional neural networks (3D CNNs) are advantageous for extracting volumetric features, they typically require consistent, high-resolution isotropic imaging protocols that were not met in our retrospective dataset. Additionally, most adrenal lesions in our cohort were visible in only a limited number of axial slices, further reducing the feasibility and potential benefit of 3D modeling. Future prospective studies using standardized isotropic MRI acquisitions may allow for more effective implementation of 3D segmentation and analysis.

Third, although we incorporated a completely unseen test data set in this work, it is based on the same institution. Our findings should also be validated with independent external data sets. Fourth, we used a single ML algorithm, namely, a support vector machine. On the other hand, we have done many experiments with different feature selection algorithms. With larger data sets, the artificial neural networks, like complex algorithms, can also be utilized in future works. Fifth, we were unable to perform parameter tuning during feature selection because we used the wrapper algorithm, which optimizes the models from the perspective of optimal feature sets with respect to the classifiers used. We used the default ML parameters provided by the software used during feature selection and then applied tuning to check for any performance improvement. It is also important to emphasize that these parameters used during feature selection are the optimal parameters of many open-source ML toolkits. While internal validation with an independent test set was performed, external validation using multi-center datasets is needed to assess the generalizability of our model.

## CONCLUSION

A combined radiomic-radiologic machine learning model based on multi-sequence MRI (T2W, in-phase, out-of-phase, and ADC sequences) might be a promising non-invasive method in differentiating fat-poor and atypical adrenal adenomas from metastases. The combined radiomic-radiologic model achieved a better predictive performance than radiomic-only and radiologic-only models did. This finding is consistent with prior multimodal modeling studies in other domains, where data fusion approaches, such as variational dynamic fusion using Gaussian mixtures, have shown superior performance over unimodal models [31]. More than four-fifths of the masses can be correctly and non-invasively diagnosed with this method. This evaluation on previously unseen test cases reflects principles of causal meta-generalization, which aim to ensure model robustness and transferability across varying clinical data domains [32]. Only less than one-fifth of the radiomic features showed excellent reproducibility, necessitating a robust reliability analysis before modeling in future works.

## Figures and Tables

**Fig. (1):**
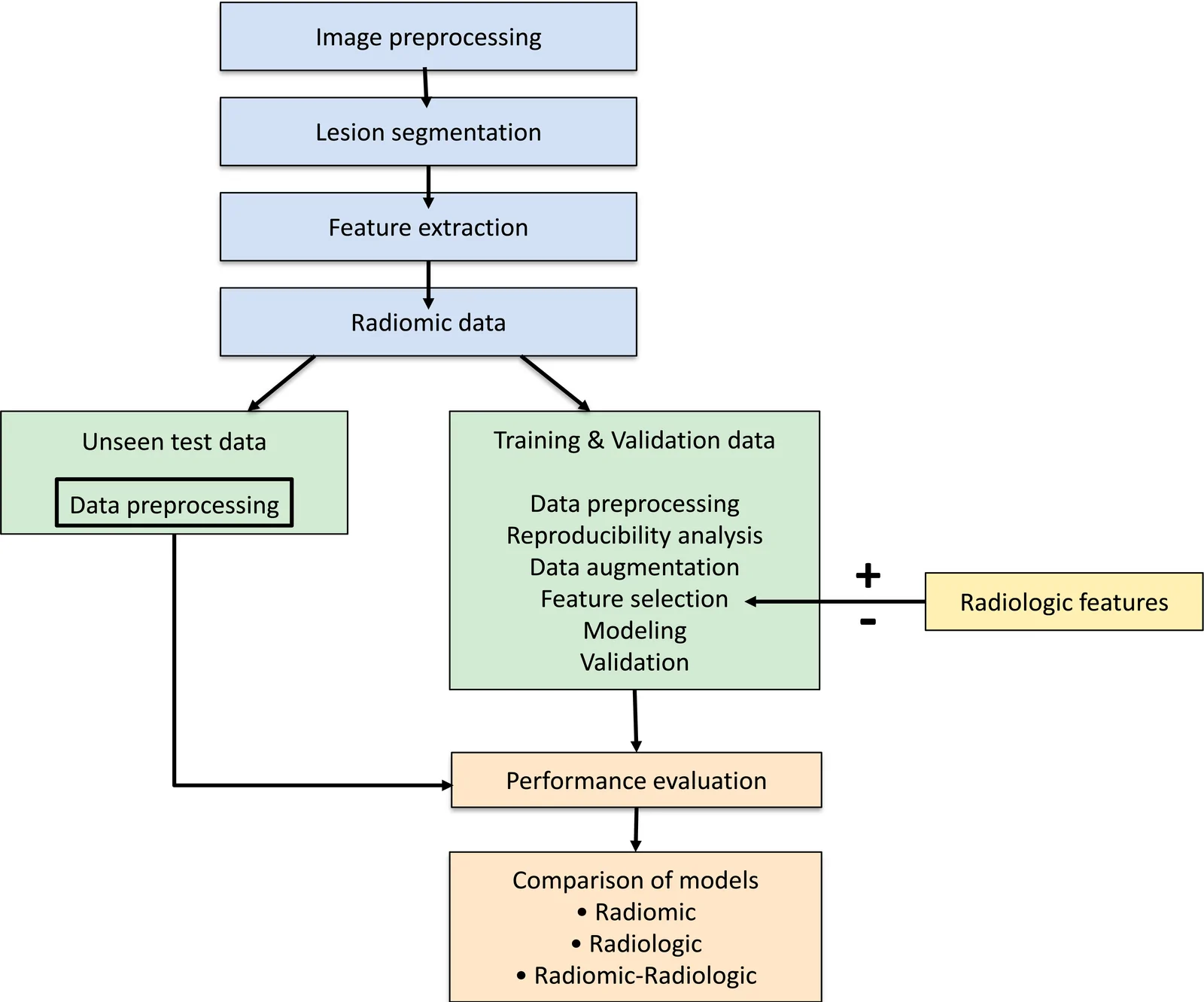
Technical study pipeline.

**Fig. (2):**
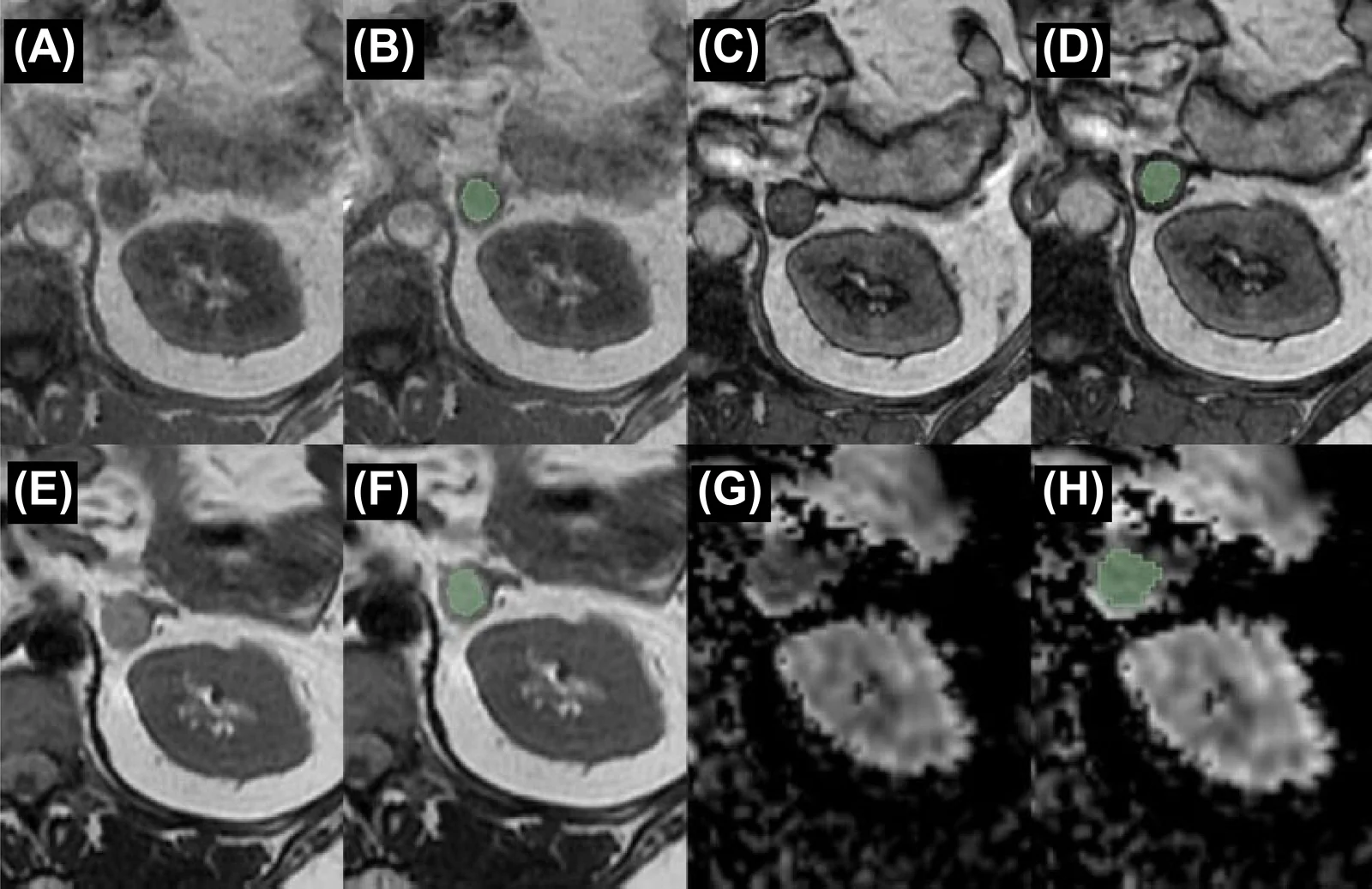
Segmentation process. In-phase chemical shift imaging (CSI) **(A, B)**, opposed-phase CSI **(C, D)**, T2-weighted image **(E, F)**, and apparent diffusion coefficient map **(G, H)**.

**Fig. (3):**
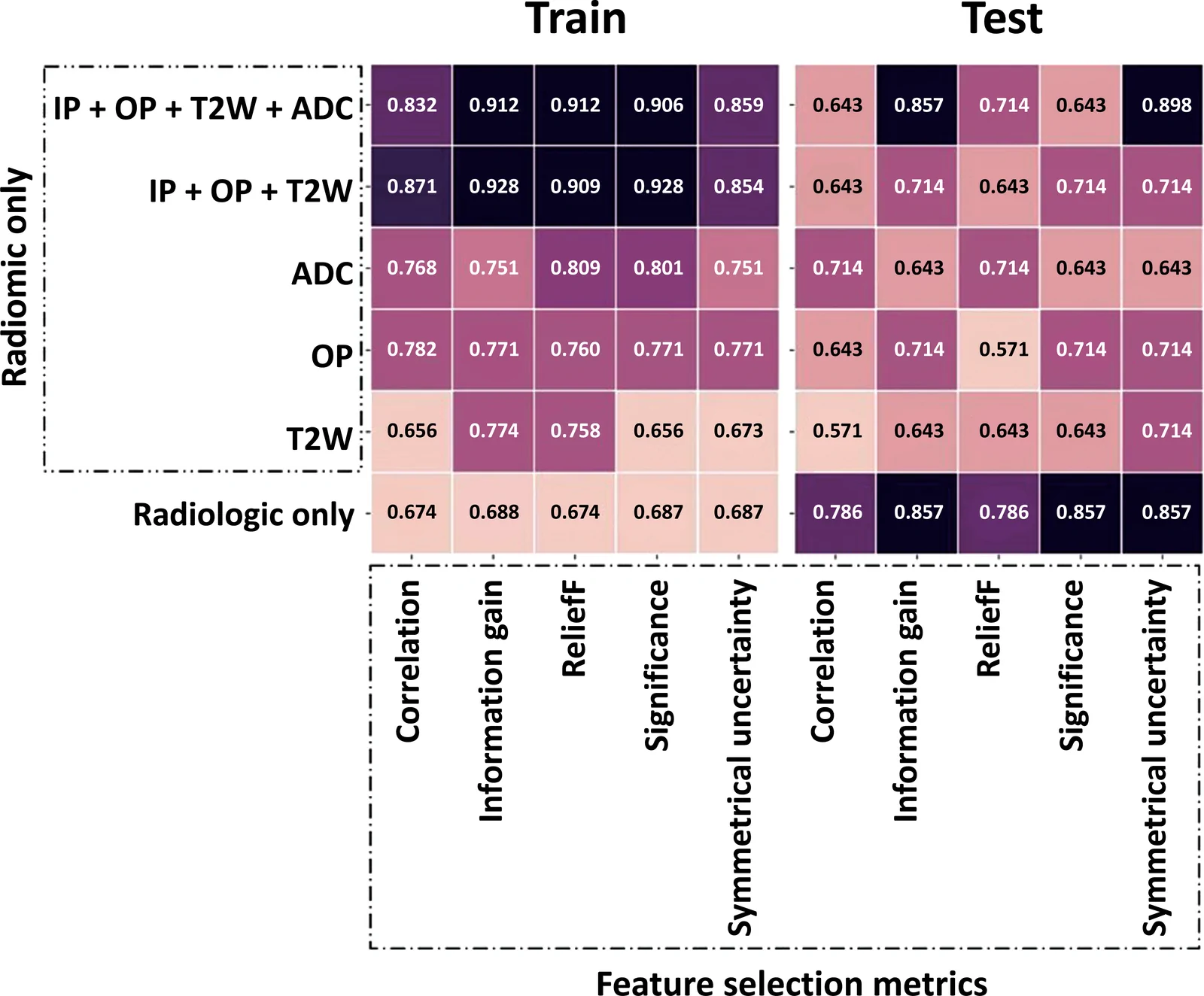
Heat-map showing the feature selection experiments done to find the best performing radiomic-only and radiologic-only machine learning models based on the area under the receiver operating characteristic curve. **Abbreviations:** (**T2W**: T2-weighted image; **OP**: opposed-phase chemical shift imaging; **IP**: in-phase chemical shift imaging; **ADC**: apparent diffusion coefficient map).

**Fig. (4):**
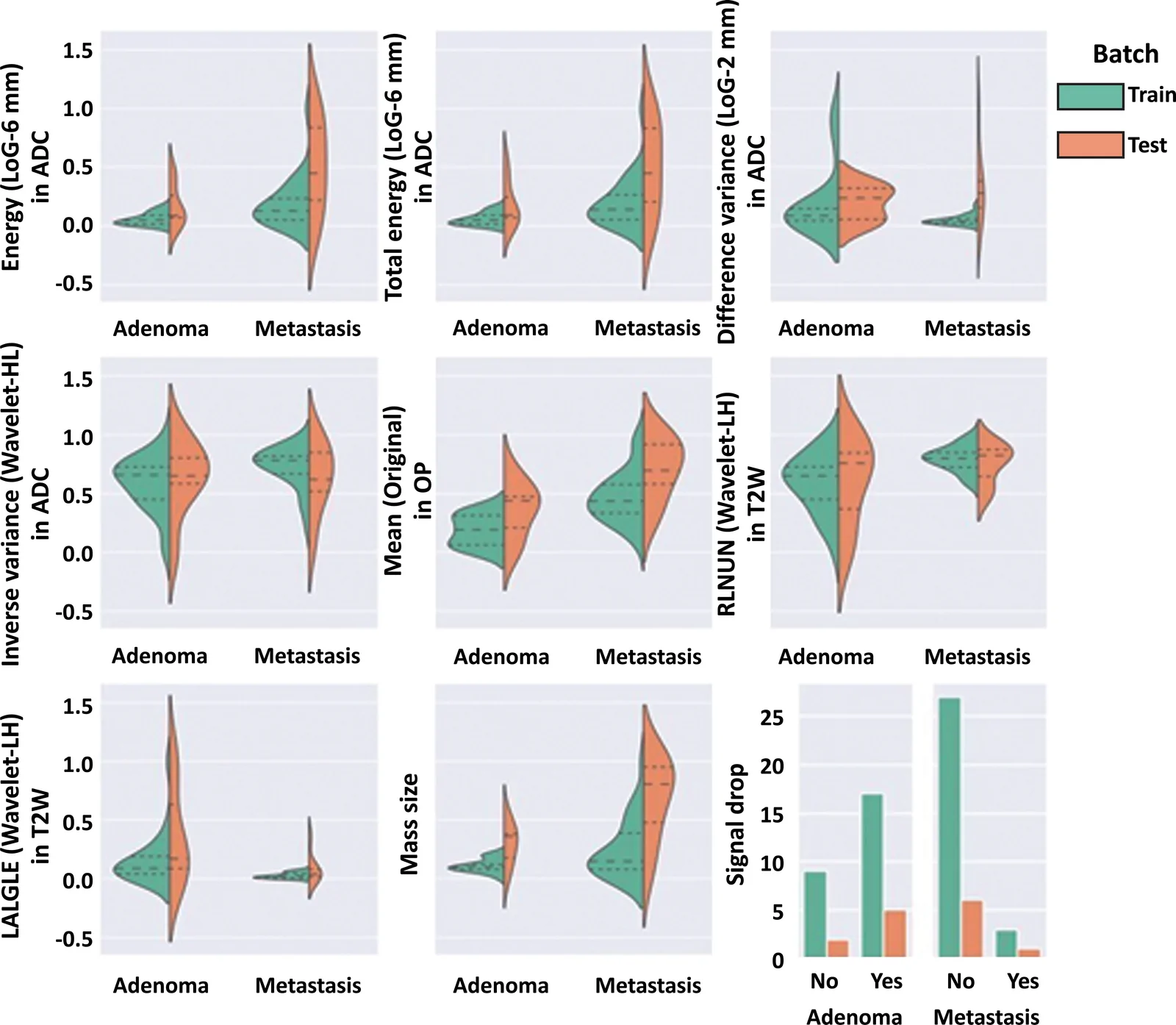
Violin plots (with median and interquartile range shown as dashed lines) and bar charts representing the distribution of the most informative features selected by the top-performing model - the radiomic-radiologic model. Each subplot compares the distribution of features between two lesion types: adenoma and metastasis, further separated into training (green) and testing (orange) batches. The X-axis labels represent lesion type (adenoma or metastasis), while the Y-axis reflects the normalized value of each selected feature. **Abbreviations: T2W** = T2-weighted, OP = out-of-phase, **ADC** = apparent diffusion coefficient, **LH** = low-high frequency band, **HL** = high-low frequency band, **LoG** = Laplacian of Gaussian.

**Fig. (5):**
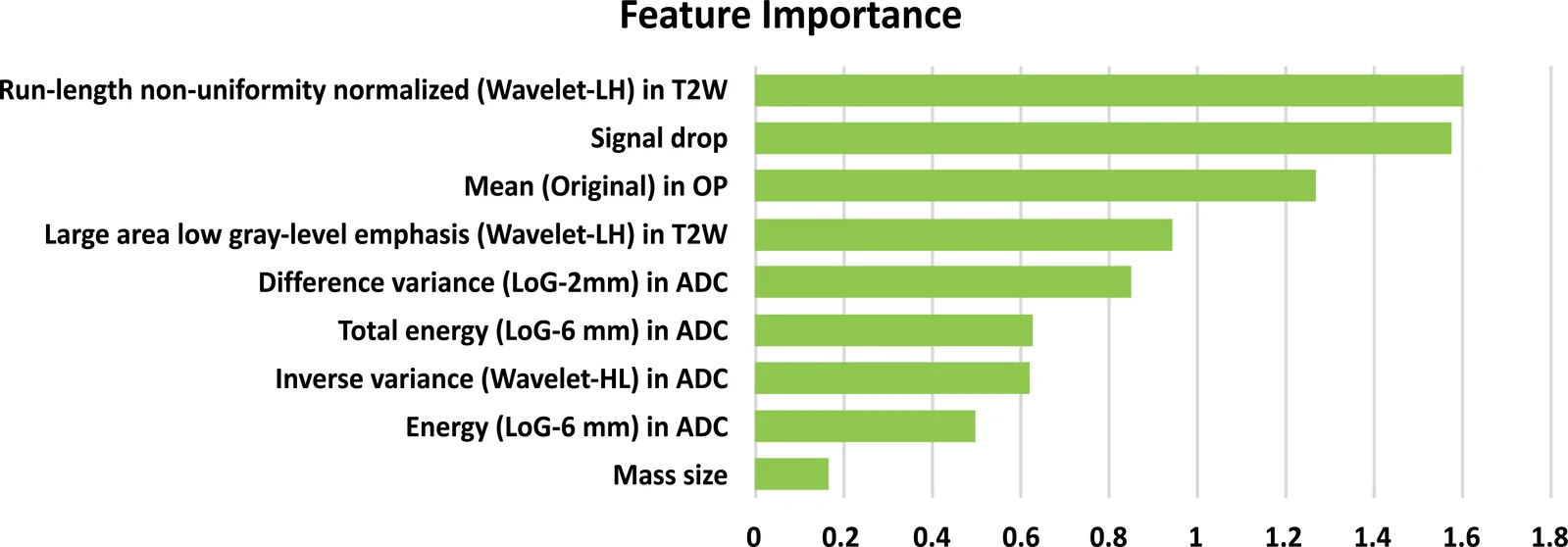
Feature importance plot of the best model, namely, the radiomic-radiologic model. Feature importance values correspond to the absolute value of coefficients in model development. Image types from which radiomic features are extracted are given in parentheses. **Abbreviations:** (**T2W**: T2-weighted image; **OP**: opposed-phase chemical shift imaging; **ADC**: apparent diffusion coefficient map; **LH**: low-high frequency band; **HL**: high-low frequency band; **LoG**: Laplacian of Gaussian).

**Fig. (6):**
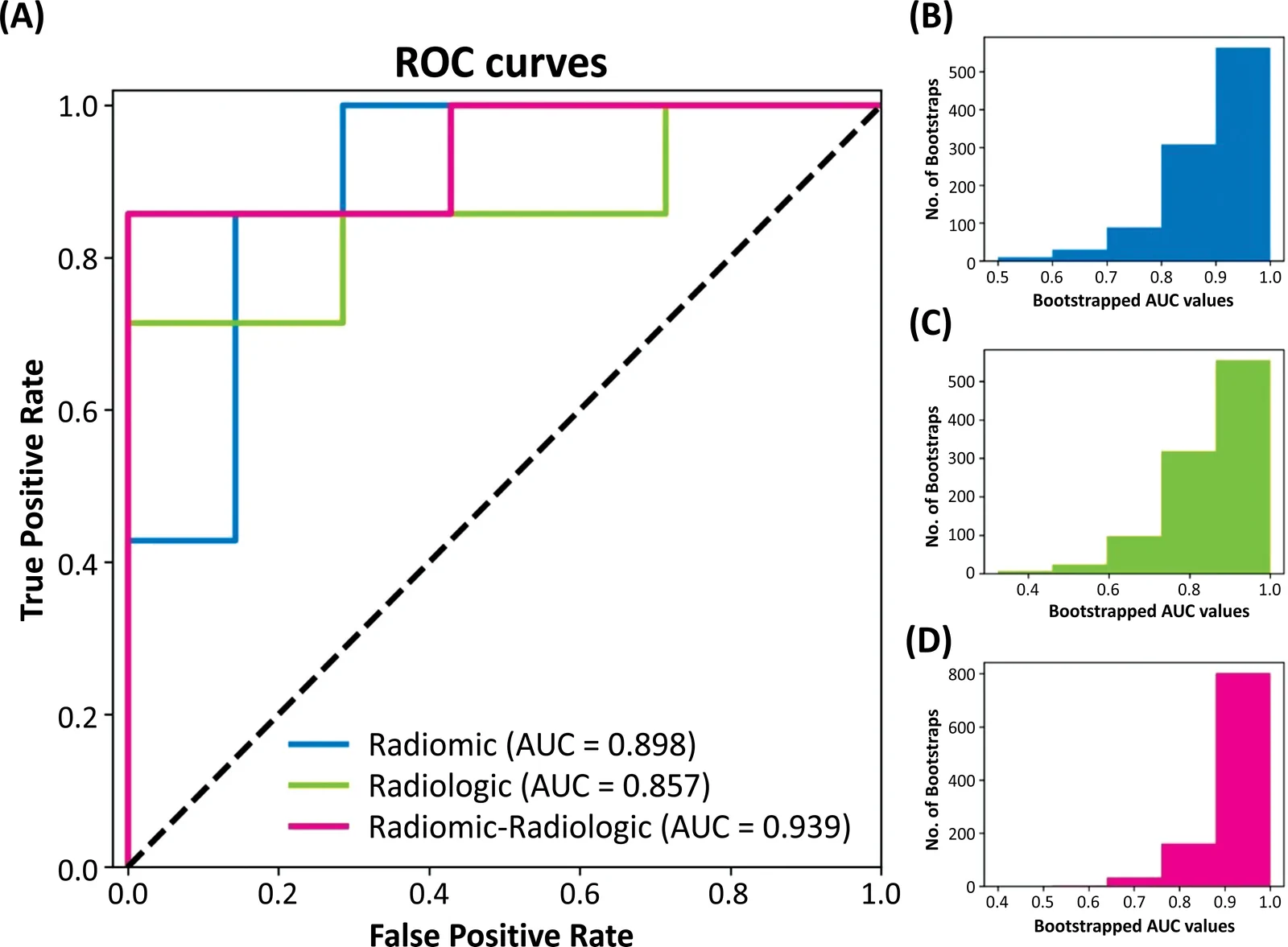
Receiver operating characteristic (ROC) curves and 1000 times bootstrapped area under the curve (AUC) values. **A**, ROC curves of the best radiomic, radiologic, and radiomic-radiologic machine learning models. **B**, Bootstrapped AUC values for radiomic only machine learning model. **C**, Bootstrapped AUC values for radiologic-only machine learning model. **D**, Bootstrapped AUC values for radiomic-radiomic machine learning model.

**Table 1:** ICC distribution.

**ICC Range**	**Number of Features**	**Percentage (%)**
ICC < 0.5	462	13.9
0.5 ≤ ICC < 0.75	1004	30.3
0.75 ≤ ICC < 0.9	1444	43.6
ICC ≥ 0.9	402	12.1
Total	3312	100

**Table 2:** Baseline patient and mass characteristics along with conventional radiologic features.

**Characteristics**	**Values (%)**
No. of patients/masses	37/44
Age (years)	61.5
Gender *Female* *Male*	18 (48.6) 19 (51.4)
Mean mass size (mm)	35.5
No. of masses in data splits *Train* *Test*	30 (68.2) 14 (31.8)
Mass types *Adenoma* *Metastasis*	19 (43.2) 25 (56.8)
Hyperintensity in T2-weighted MRI *Adenoma* *Metastasis*	4 (15.4) 22 (84.6)
Heterogeneity* Adenoma *Metastasis*	15 (41.7) 21 (58.3)
Signal drop *Adenoma* *Metastasis*	13 (18.8) 3 (81.2)
Labeled slices *Train* *Test*	56 (80.0) 14 (20.0)

Note: *As assessed in in-phase, out-of-phase or T2-weighted images.

**Table 3:** Selected radiomic and radiologic features for the best-performing machine learning models.

**Feature origins**	**Feature Name (MRI sequence)**	**Feature Class**	**Image Type**
Radiomic	Mean (OP)	First-order	Original
	Run-length non-uniformity normalized (T2W)	GLRLM	Wavelet (Low-High)
	Large area low gray-level emphasis (T2W)	GLSZM	Wavelet (Low-High)
	Total energy (ADC)	First-order	LoG filter of 6 mm
	Energy (ADC)	First-order	LoG filter of 6 mm
	Inverse variance (ADC)	GLCM	Wavelet (High-Low)
	Difference variance (ADC)	GLCM	LoG filter of 2 mm
Radiologic	Mass size	-	-
	Signal drop	-	-

**Note:** T2W = T2-weighted, OP = out-of-phase, ADC = apparent diffusion coefficient, GLRLM = gray-level run-length matrix, GLSZM = gray-level size-zone matrix, GLCM = gray-level co-occurrence matrix, LoG = Laplacian of Gaussian.

**Table 4:** Best performing machine learning models with 95% confidence intervals.

Model	Accuracy (%)	Sensitivity (%)	Specificity (%)	AUC
Train (10-fold CV)			
Radiomic	85.7 (79.2–91.0)	88.5 (79.2–95.0)	83.3 (71.4–91.7)	0.859 (0.781–0.918)
Radiologic	78.5 (71.2–85.4)	65.4 (53.3–76.1)	90.0 (78.2–96.7)	0.687 (0.590–0.776)
Radiomic-Radiologic	85.7 (79.2–91.0)	80.8 (70.2–89.0)	90.0 (78.2–96.7)	0.965 (0.918–0.990)
Test			
Radiomic	85.7 (63.7–97.0)	71.4 (41.9–91.6)	100 (73.5–100)	0.898 (0.708–1.000)
Radiologic	78.6 (55.5–93.7)	71.4 (41.9–91.6)	85.7 (57.2–98.2)	0.857 (0.640–0.984)
Radiomic-Radiologic	85.7 (63.7–97.0)	85.7 (57.2–98.2)	85.7 (57.2–98.2)	0.939 (0.746–0.997)

**Note:** For sensitivity and specificity, the first and second rows indicate the results for adenoma and metastasis classes, respectively. AUC, area under the curve.

## Data Availability

The authors confirm that the data supporting the findings of this research are available within the article.

## LIST OF ABBREVIATIONS

ADC: Apparent Diffusion Coefficient
AUC: Area Under the Curve
FOV: Field of View
GLCM: Gray-Level Co-occurrence Matrix
GLRLM: Gray-Level Run-Length Matrix
GLSZM: Gray-Level Size-Zone Matrix
LoG: Laplacian of Gaussian
ML: Machine Learning
NEX: Number of Excitations
NGTDM: Neighbouring Gray Tone Difference Matrix
OP: Out-of-Phase
RLNU: Run-Length Non-Uniformity
ROC: Receiver Operating Characteristic
TE: Echo Time
TR: Repetition Time
T2W: T2-Weighted Imaging

## STANDARDS OF REPORTING

STROBE guidelines were followed.
