# Supplementary material for: Differentiation of Fat-poor and Atypical Adrenal Adenomas from Metastases: MRI-based Radiomic, Radiologic, and Radiomic-radiologic Machine Learning Models
Source: Curr Med Imaging. 2026 Apr 8;22:E15734056399745. doi: 10.2174/0115734056399745260330003149 (PMC13579555; doi:10.2174/0115734056399745260330003149)
Supplement: Supplementary file 1 [file CMIM-22-E15734056399745_SD1.pdf]

1875-6603/26

Send Orders for Reprints to reprints@benthamscience.net

BENTHAM SCIENCE

CrossMark

Current Medical Imaging

Content list available at: https://benthamscience.com/journals/cmir

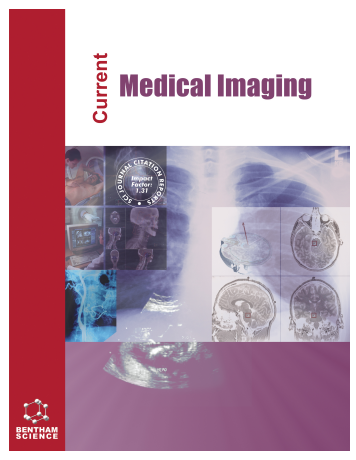

Supplementary Material

Differentiation of Fat-poor and Atypical Adrenal Adenomas from Metastases: MRI-based Radiomic, Radiologic, and Radiomic-radiologic Machine Learning Models

Ceyda Turan Bektaş[1,*], Hasan Bulut[2], Ece Ateş Kuş[3], Melis Baykara Ulusan[4] and Abdullah Soydan Mahmutoğlu[1]

[1]*Department of Radiology, Istanbul Training and Research Hospital, Istanbul, Türkiye*
[2]*Department of Radiology, Ankara Etlik City Hospital, Ankara, Türkiye*
[3]*Department of Diagnostic and Interventional Radiology, Medical Faculty OWL, University of Bielefeld, Klinikum Lippe, Bielefeld, Germany*
[4]*Nottingham Breast Institute, Nottingham University Hospitals, NHS Trust, Nottingham, UK*

Part A

Feature Selection Experiments

The correlation method assesses the worth of a feature by quantifying Pearson's correlation between the feature and the respective classes. Information gain assesses the worth of a feature by quantifying the information gain concerning the classes, without considering feature interactions. ReliefF ranks the features according to k-nearest neighbors. The significance method assesses the features by calculating the probabilistic significance as a two-way function. The symmetric uncertainty technique assesses the feature importance by calculating the symmetrical uncertainty related to another set of features.

Part B

MRI Technique

The acquisition parameters for the OP-CSI sequence were as follows: repetition time/ echo time (TR/TE), 115-255/2.01-2.25 msec; field of view (FOV), 780x380-900x440 mm; slice thickness, 5.5-7 mm; and the number of excitations (NEX), 1. The parameters of IP-CSI were as follows: TR/TE, 130-255/4.2-4.81 msec; FOV, 780x380-900x440 mm; slice thickness, 5.5-7 mm; and NEX, 1. For T2W sequence, they were as follows: TR/TE, 613-905/87-91 msec; FOV, 780x380-900x440 mm; slice thickness, 5.5-7 mm; and NEX, 0.55. For DWI sequence, those were as follows: TR/TE, 5425-6025/64-66 msec; FOV, 780x380-900x440 mm; slice thickness, 5.5-7 mm; and NEX, 1-2. ADC maps for each lesion were generated from DWI obtained using b0 s/mm2 and b800 s/mm2 values.

© 2026 The Author(s). Published by Bentham Science Publisher.

This is an open access article distributed under the terms of the Creative Commons Attribution 4.0 International Public License (CC-BY 4.0), a copy of which is available at: https://creativecommons.org/licenses/by/4.0/legalcode. This license permits unrestricted use, distribution, and reproduction in any medium, provided the original author and source are credited.

HOW TO CITE:
Bektaş C, Bulut H, Kuş E, Ulusan M, Mahmutoğlu A. Differentiation of Fat-poor and Atypical Adrenal Adenomas from Metastases: MRI-based Radiomic, Radiologic, and Radiomic-radiologic Machine Learning Models. Curr Med Imaging, 2026; 22: e15734056399745. http://dx.doi.org/10.2174/0115734056399745260330003149
